# Integrating 1D-CNN and Bi-GRU for ENF-Based Video Tampering Detection

**DOI:** 10.3390/s25216612

**Published:** 2025-10-28

**Authors:** Xiaodan Lin, Xinhuan Zang

**Affiliations:** School of Information Science and Engineering, Huaqiao University, Xiamen 361021, China

**Keywords:** electric network frequency, inter-frame tampering, tampering detection, CMOS sensors

## Abstract

Electric network frequency (ENF) refers to the transmission frequency of a power grid, which fluctuates around 50 Hz or 60 Hz. Videos captured in a power grid environment may exhibit flickering artifact caused by the intensity variation in the light source, thus exhibiting the flickering pattern according to the ENF fluctuation. This flicker, notable for its temporal dynamics and quasi-periodic property, acts as an effective means for video tampering forensics. However, ground-truth ENF databases are often unavailable in a real-world authentication setting, thus posing challenges in conducting ENF examination in video forensics. In addition, dynamic scenes in videos also increase the difficulty of anomaly detection in ENF signals. To address these challenges, we proposed an approach based on neural networks to detect inter-frame tampering in CMOS videos that incorporate ENF signals. To the best of our knowledge, this is the first work that deploys data-driven approach for ENF-based video forensics. Without the aid of the reference ENF dataset, we exploited the implicit ENF variation in luminance signals and transformed the video signal into a one-dimensional time series utilizing ENF priors. In addition, to alleviate the impact of moving objects that also cause the variation in luminance signal, a preprocessing stage is proposed. On this basis, we designed an anomaly detection model combining 1D-CNN and Bi-GRU to conduct experiments on static and dynamic video datasets. The experimental results demonstrate the effectiveness of our proposed method in inter-frame video tampering detection, implying its potential as a forensic tool for ENF-based video analysis.

## 1. Introduction

With the evolution of the internet, images and videos have become prevalent means of communication. Given the substantial information carried within, these media formats have become prime targets for malicious attacks and dissemination on social media platforms. Meanwhile, the rapid development in multimedia forensics has led to numerous forensic methodologies over recent years.

Electric network frequency (ENF) is the transmission frequency of the power grid, which has different nominal values in different regions, e.g., 50 Hz in China, Australia, and other regions, and 60 Hz in North America. According to previous studies [1,2], ENF signal was first discovered in audio files. Researchers have shown that ENF is a temporally varying signal, exhibiting uniform variations within the same electric grid. Due to its unique and stationary characteristics, ENF signal has been widely employed in audio forensics. For example, studies [3,4,5] demonstrated that ENF signal can be used to achieve time of recording verification, and the studies in [6,7,8] proved that ENF signals can enable location authentication. In addition, ENF signal was also utilized as a tool for audio tampering detection [9,10,11,12,13]. Furthermore, the potential of ENF signals as physical watermarks to defend multimedia data against deepfake attack is explored in [14,15].

Recently, studies [16,17,18,19,20,21,22,23] have shown that ENF signal can also be extracted from image and video recordings under mains power lighting. This is because light intensity fluctuations in LED and incandescent bulbs produce flickers imperceptible to the human eye but can be captured by recording devices at a frequency of twice the nominal ENF. Therefore, video forensics based on ENF signal becomes feasible. Earlier studies [17,24] extracted ENF signals from videos and then compared them with ENF reference databases to identify and locate tampered video. A follow-up study [25] achieved blind detection of CCD videos without reference by using the relationship between the correlation coefficients between signals in adjacent cycles of an ENF signal. Although these studies have proved that ENF signals can be used for video forensics, there are still some limitations. Firstly, it is hard to estimate highly accurate ENF signals, especially for dynamic scenes. Secondly, the real-time reference ENF database is often unavailable.

To address the above limitations, a data-driven approach based on lightweight CNN combined with recurrent neural networks is proposed to detect inter-frame tampering in CMOS videos where ENF signals are implicitly embedded. This approach is the first attempt to deploy neural networks for ENF-based video forensic research without the aid of reference signals.

Our main contributions are as follows:

- Aiming at the deficiency of video data, we collected static scene videos and dynamic scene videos from studies [22,26,27], which were captured in different regions and covered two nominal ENF frequencies. Then we generated several tampered video datasets using FFMPEG for training and testing, resulting in three types of inter-frame tampering videos, i.e., frame deletion, frame duplication, and frame insertion.
- Without the need of extracting ENF signals, the proposed method can directly leverage luminance information for tamper detection. Particularly, we employed the Gaussian Mixture Model for background subtraction to identify and mask moving objects of each frame to reduce the interference of moving scenes with illumination signal.
- We designed a lightweight but efficient model integrating one-dimensional convolutional neural network (1D-CNN) and Bidirectional Gated Recurrent Unit (Bi-GRU) for anomaly detection in luminance signals of videos, which can learn local features and temporal dependencies of input sequences and distinguish whether videos have been tampered or not.

The rest of this paper is structured as follows: Section 2 reviews ENF-based video forensic works. Section 3 describes our proposed method. Section 4 presents the experimental results. Finally, we provide the conclusion in Section 5.

## 2. Related Work

In recent years, there has been a number of research endeavors in the domain of ENF-based video forensics. To be specific, authors performed insertion and deletion operations on videos, and compared the extracted ENF signals with a reference ENF database to identify where the video was edited [17]. In [24], the authors proposed the Selective Superpixel Masking method to compensate for occlusions attributed to moving objects and then extracted accurate ENF signals to compare with a reference ground-truth database to authenticate whether a video was forged or not.

The study in [25] investigated the implementation of video inter-frame tampering detection and localisation in CCD videos with ENF signals. The authors observed that inter-frame tampering causes the correlation coefficient of the ENF signal in the neighbouring cycles to be significantly smaller. Therefore, the authors relied on the abrupt decrease in the correlation coefficient to determine the presence and location of tampering, which overcomes the limitation of relying on a reference ENF database, but the experimental dataset only contained CCD videos under 50 Hz grid, and the accuracy was still far from satisfaction.

Recently, a method for detecting deepfakes in online video conferencing platforms named DeFakePro was proposed in [28] using a decentralized consensus mechanism. This technique utilized the ENF present in digital media recordings as a unique environmental signature, which was used in the proof-of-ENF (PoENF) algorithm to establish a consensus mechanism. The PoENF algorithm compared the variations in the ENF signal to validate media broadcasts on conferencing platforms. Although the work proved that ENF signals can be employed for deepfaked video forensics, reference ENF data is still needed.

The above studies mostly require extracting accurate ENF signals and identifying the authenticity of the videos with the aid of reference data. However, it is hard to get access to the reference ENF databases in most real-world situations. To address these challenges, we proposed a method to enable ENF-based video tampering detection without leveraging reference ENF signals.

## 3. The Proposed Method

**Figure 1:**
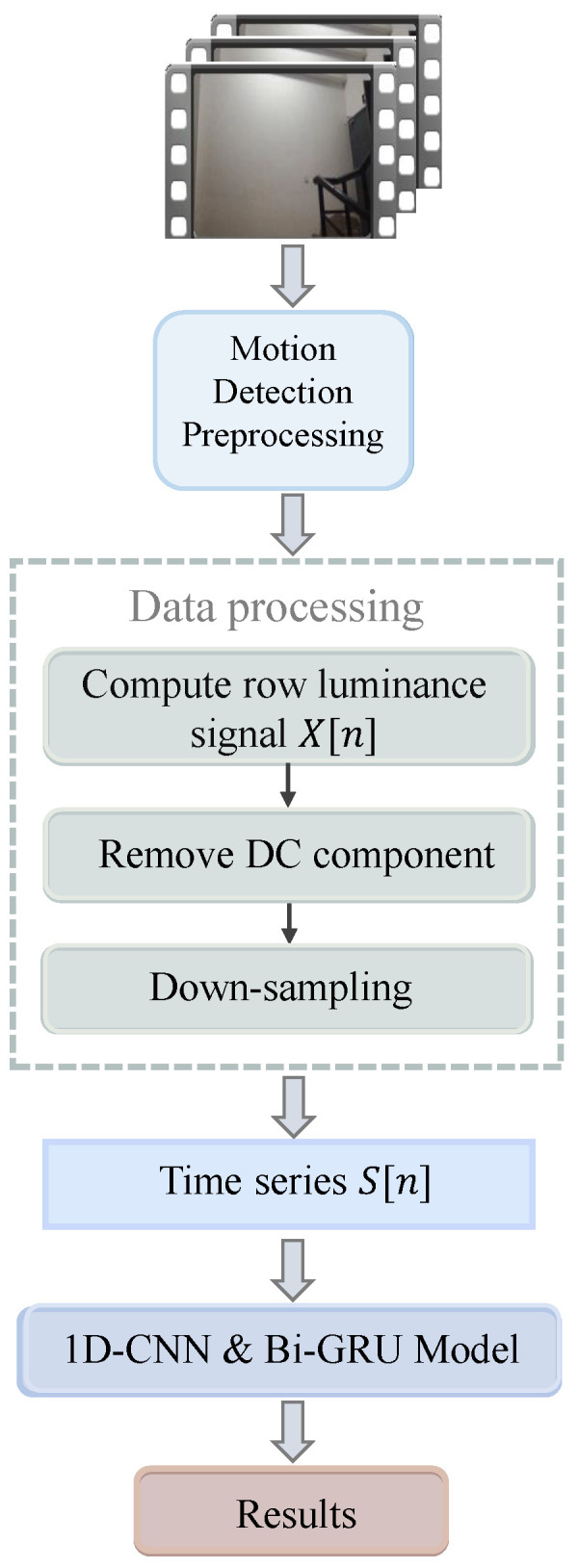
The framework of video tampering detection.

In the following, we will introduce the proposed method for inter-frame tampering forensics of video under rolling shutter exposure. Essentially, the motivation of this work inspired from the effect of video tampering on ENF signals is also presented in this section. The framework of the proposed system is shown in Figure 1, where an extra preprocessing step is required for dynamic videos.

### 3.1. The Effect of Video Tampering on ENF Signals

**Figure 2:**
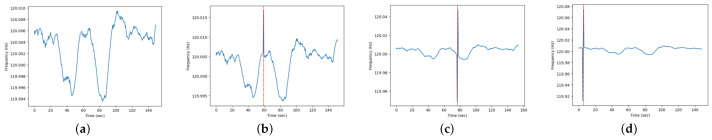
An example of the ENF signals for original and tampered static videos: (**a**) ENF signal from original video; (**b**) ENF signal from frame-deletion video; (**c**) ENF signal from frame-duplication video; (**d**) ENF signal from frame-insertion video.

Video tampering is often performed carefully to avoid the perception by human eyes. However, weak ENF signals captured by CMOS cameras can show telltale signs of video tampering. Following the method described in [22], we extracted ENF signals from a static video and three tampered videos to compare the difference in ENF signals before and after video tampering. An example of the ENF signals of the original static video and three tampered videos is shown in Figure 2, where the ground-truth tampering location is marked with a red dash line. The original video has a frame rate of 25 and its length is 150 s, captured in an electric grid with the nominal value of 60 Hz. In Figure 2b, a video segment of 1 s was deleted from the original video at the position of about 60 s. In Figure 2c, a duplicated video segment of one-second length was placed at 77 s. And in Figure 2d, frame insertion was conducted at approximately the second of 6.

It can be noticed that the three types of tampering all cause abnormal deviations in the ENF signal in static scenes. Theoretically, video tampering can be identified by comparing the estimated ENF signal with a threshold, but it is hard to determine the accurate threshold. Moreover, things become more complicated when dealing with dynamic videos, since ENF estimation is susceptible to interference from dynamic scenes, which can be observed in Figure 4.

### 3.2. Dynamic Video Preprocessing

**Figure 3:**
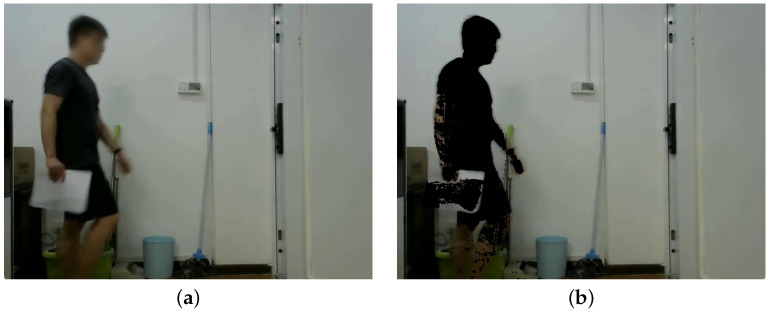
An example of a dynamic video before and after motion detection preprocessing: (**a**) original video; (**b**) preprocessed video.

Since the ENF variation is contained in the luminance signal of videos, it is necessary to consider the interference of object motion that can have an influence on the luminance signals. Background subtraction based on Gaussian Mixture Model was utilized to mask the moving objects [29], so as to alleviate the impact of motion on luminance. Subsequently, a foreground mask for each frame was obtained after conducting background substraction. This mask was binarized to distinguish moving objects from the background. An OpenCV implementation (version 4.9.0) was made for background substraction in this work, with the number of frames used to build the background model being 150 and a threshold value of 70. As the example shown in Figure 3, the moving objects were marked as black and the pixels in static regions remain unchanged.

**Figure 4:**
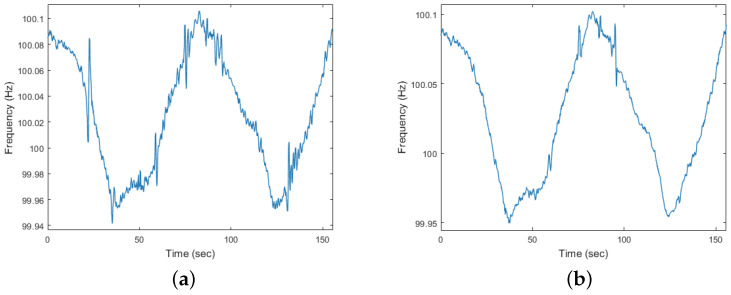
An example of the ENF signals extracted from a dynamic video before and after motion detection preprocessing: (**a**) ENF signal from original video; (**b**) ENF signal from preprocessed video.

To illustrate the interference from dynamic scenes for ENF extraction, Figure 4 presents the results of the ENF signals before and after motion detection preprocessing of a dynamic video that is not tampered. The video was captured in an electric grid with the nominal value of 50 Hz. As can be seen in Figure 4a, the ENF signal extracted without preprocessing fluctuates a lot and exhibits abnormal abrupt values. On the contrary, the ENF signal in Figure 4b appears to be more smooth, implying that the impact of dynamic scenes is much alleviated. Therefore, this preprocessing stage for dynamic videos greatly avoids the interference from moving objects on luminance signal.

### 3.3. Processing of Illumination Signal

Subsequently, we proceeded to investigate the luminance signal of the video instead of the ENF signal, since the ENF signal is also contained in the luminance but still needs to be estimated. The ENF signal captured by rolling shutters is sampled sequentially in a rowwise manner, so we calculated the average luminance of each row within a frame and concatenated the results across all the frames, thereby producing time series data referred to as the row luminance signal S[n]. Specifically, the average luminance was computed row by row and concatenated into a unique time series X[n] at a sampling rate of $f_{s}=f_{c}*n_{row}$, where $f_{c}$ refers to the video frame rate and $n_{row}$ refers to the height of the video. We then applied two processing steps to the time series:

- Remove DC component: The time series X[n] removes its DC component by subtracting mean luminance.
- Down-sampling: Downsample the time series X[n] after subtracting average luminance to 1 kHz to get a new time series S[n] for the input to the model as the sampling rate of row luminance signal is much higher than the underlying ENF.

**Figure 5:**
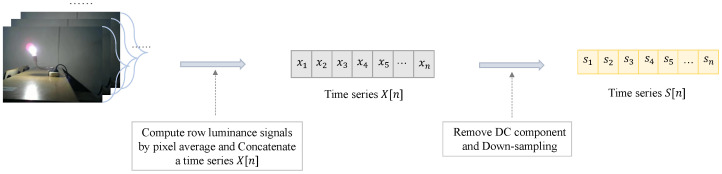
DC component removal and downsampling after capturing the row luminance signal from video frames.

Figure 5 demonstrates the process of using luminance information to transform the video signal into time series S[n]. For static videos, we can directly apply the above two steps to process row luminance signal. For dynamic videos, it is necessary to apply the preprocessing method proposed in the previous subsection. Specifically, we only computed the average luminance of the unmasked pixels in each row when acquiring X[n]. After iterating through all the frames, time series S[n] was yielded as the input of the neural network model.

### 3.4. Network Model Based on 1D-CNN & Bi-GRU

**Figure 6:**
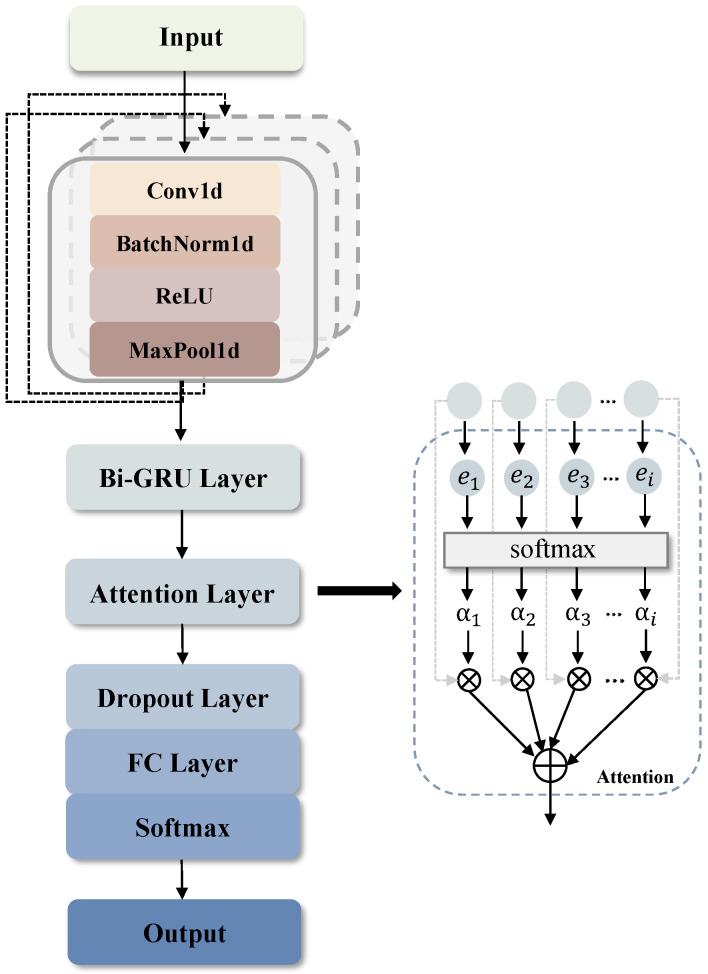
The structure of the proposed tampering detection model.

From the previous section, we have prepared one dimensional time series data, hence the video tampering detection can be converted into a time series anomaly detection task. As inter-frame video tampering usually occurs in limited time spans, a convolutional block employed to extract the local features from the time series data thanks to its ability in capturing local and global context. As demonstrated in the previous section, the abnormal variation in the row luminance signal can be an indicator for potential video tampering. Particularly, the interdependence in the row luminance signal is essential to identify the anomalies. The recurrent neural network [30] boasting of recurrent mechanism has been proved to be competent in capturing the dynamic temperal relationship in sequential signals; it is, therefore, employed in this work for amomaly detection. To be specific, one-dimensional convolutional neural network (1D-CNN) and Bidirectional Gated Recurrent Unit (Bi-GRU) [31] were applied to build the basic framework, using the preprocessed time series S[n] as the input. Further, a self-attention layer [32] was added to enhance the feature representation learning. Figure 6 presents the structure of our proposed anomaly detection model, whose detailed configurations are listed in Table 1. Note that each Conv Block in Table 1 includes 1D convolution, batch normalization, ReLU and 1D Maxpooling.

Specifically, the input sequence S[n] was initially processed through three convolutional blocks, which can be represented as

(1) $$H_{\mathrm{CNN}}={\mathrm{CNN}\_\mathrm{Block}}_{3}\left({\mathrm{CNN}\_\mathrm{Block}}_{2}\left({\mathrm{CNN}\_\mathrm{Block}}_{1}\left(S\left[n\right]\right)\right)\right)$$

where all CNN_Block contain a convolutional layer, a batch normalization layer, a ReLU activation function, and a max pooling layer. Within each convolutional block, the size of the convolution kernel was set to 3, the stride and padding were both set to 1. The output $H_{\mathrm{CNN}}$ had a shape of (B,L,C), where *B* is the batch size, *L* represents the sequence length, and *C* indicates the number of channels, which is set to 128 in the experiment.

The output $H_{\mathrm{CNN}}$ was subsequently fed to the Bi-GRU layer:

(2) $$H_{\mathrm{Bi}\text{-}\mathrm{GRU}}=\mathrm{Bi}\text{-}\mathrm{GRU}\left(H_{\mathrm{CNN}}\right)$$

The resulting output, $H_{\mathrm{Bi}\text{-}\mathrm{GRU}}$, had dimensions $\left(B,L^{\prime },2H\right)$, where $L^{\prime }$ is the new sequence length after processing by the Bi-GRU, and 2H represents the dimensionality of the hidden states.

For the attention layer, firstly we computed scores by applying a linear layer to $H_{\mathrm{Bi}\text{-}\mathrm{GRU}}$. The score matrix E for the entire sequence was computed as follows:

(3) $$E=H_{\mathrm{Bi}\text{-}\mathrm{GRU}}W_{e}+b_{e}$$

Here, E had a shape of $\left(B,L^{\prime }\right)$, $W_{e}$ is the weight matrix with dimensions (2H,1), and $b_{e}$ is a scalar bias term.

Next, the softmax function was applied to the score matrix E to derive the attention weights α for the entire sequence. Subsequently, we computed the weighted average of $H_{\mathrm{Bi}\text{-}\mathrm{GRU}}$ using α to obtain the context vector C:

(4) $$C=\alpha H_{\mathrm{Bi}\text{-}\mathrm{GRU}}$$

where C has dimensions (B,2H). To prevent overfitting, a dropout layer is introduced following the attention layer. The dropout ratio is set to 0.5.

Finally, $C_{\mathrm{dropout}}$ was passed through a fully connected layer followed by softmax activation function to obtain the classification probabilities y, which indicated whether the sequence was normal or abnormal:

(5) $$y=\mathrm{softmax}(W_{o}C_{\mathrm{dropout}}+b_{o})$$

where $W_{o}$ is the weight matrix of the fully connected layer with shape (2H,2), and $b_{o}$ is the bias vector.

## 4. Experiments

### 4.1. Datasets and Experimental Settings

**Figure 7:**
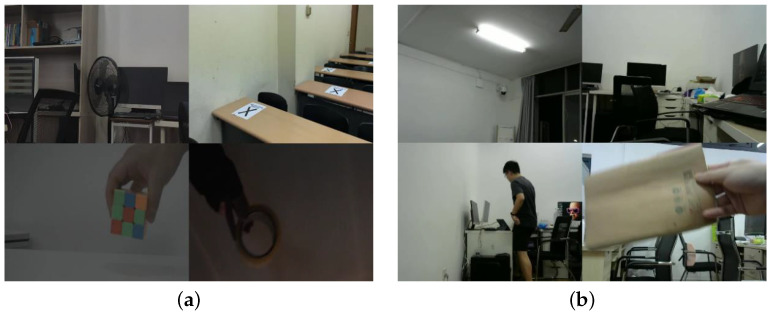
Sample videos from the datasets: (**a**) sample video scenes from Dataset-1; (**b**) sample video scenes from Dataset-2.

We produced two video datasets, named Dataset-1 and Dataset-2, covering different scenes and two nominal ENF frequencies, where Dataset-1 was used for training based on publicly available video data in [22,27], and Dataset-2, originating from [26], was used for testing. In Dataset-1, dynamic videos were recorded at power grids with a normial ENF of 50 Hz and static videos were recorded at 50 Hz and 60 Hz grids. In Dataset-2, all the videos were captured under the 50 Hz power grid. The two datasets were collected from different geographical regions, ensuring that the training set and the test set are completely disjoint. Each dataset contains equal number of static scene and dynamic scene videos. Some exemplary video scenes are provided in Figure 7.

Raw videos were cropped to about one-minute length using FFMPEG [33] to compose the positive samples. Then, we conducted three tampering operations on the videos using FFMPEG to compose negative samples, i.e., frame deletion, frame duplication, and frame insertion. The tampering operations were executed randomly from the timescale. For instance, three frames were randomly chosen for deletion, which could correspond to the 10th, 401th, and 923th frame. The ratio of normal to tampered videos in each dataset was 1:1. Other details of the datasets are shown in Table 2.

The model was trained using the binary cross entropy loss with Adam optimizer for 100 epochs. The learning rate was 1.0 × $10^{-4}$ and the batch size was 16. In the experiments, the metric of accuracy was employed to evaluate the proposed scheme.

### 4.2. Experimental Results and Discussions

In order to verify the effectiveness of the proposed design, we conducted multiple sets of experiments on the cross-domain video datasets.

**Figure 8:**
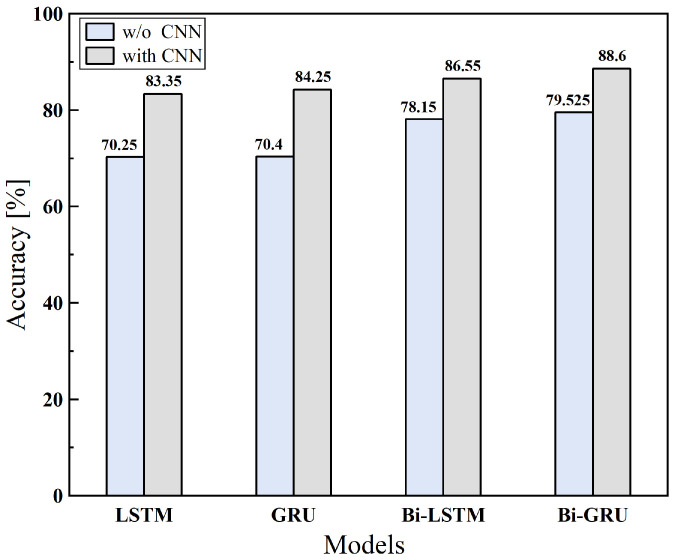
The performance of different RNN models.

Firstly, we verified the superiority of the backbone of our proposed model, i.e., Bi-GRU in conjunction with CNN. Note that these tests were carried out in the absence of the attention layer of Figure 6. In the first phrase, three pure recurrent structures, i.e., LSTM [34], Bi-LSTM [35], and GRU, were compared. The results are shown in Figure 8. As the results suggest, the Bi-GRU structure delivers the best performance among them. In the second phrase, the benefit of integrating the CNN into the above recurrent structures was demonstrated. As Figure 8 suggests, the bidirectional model is superior than the unidirectional ones like LSTM and GRU, while the Bi-GRU model is the most efficient. Since the bidirectional model is capable of simultaneously considering both the past and future data points in the sequence, it yields more robust representation. This is vital for our given task, which relies on the local anomaly of the row luminance signal. Noteworthily, compared to the Bi-LSTM model, which ranks second, the Bi-GRU model boasts a simpler architecture and fewer parameters without compromising the detection performance. Therefore, Bi-GRU generalizes better than Bi-LSTM, given the limited amount of training data in our study case. Further, the breakdown result on the test set for three types of tampering was studied, with an accuracy of 91.3%, 94.6%, and 95.4% for frame deletion, frame duplication, and frame insertion. This result shows that the proposed scheme is more effecitive on tampering types like frame duplication and frame insertion because these two tampering types usually result in more obvious ENF variation, which is consistent with the observation from Figure 2.

To verify the effect of the attention mechanism, the following ablation study was made. The performance was assessed by adding the attention layer in Figure 6 to the best performing model shown in Figure 8, dubbed CNN-Bi-GRU-Attention model. Correspondingly, the model without attention layer is named CNN-Bi-GRU. The results are shown in Table 3. It is evident that the attention mechanism has a very positive influence on the performance, increasing the accuracy from 88.6% to 93.75%. The core principle of the attention mechanism is to assign weights to the extracted features from Bi-GRU layer based on its significance, allowing the underlying model to concentrate on more discriminative information. By introducing the attention mechanism, the model is better at handling delicate input patterns and thus significantly boosts the performance. Moreover, the training expenditure of CNN-Bi-GRU-Attention model is comparable to the basic model.

Since the ENF signal is implicitly contained in the extracted row luminance signal, it reasonable to perform video forgery detection given the ENF as the input of the framework. In the following, the comparison of different input was made, i.e., the ENF signal and the row luminance signal were used as the input of the proposed CNN-Bi-GRU-Attention model seperately. The state-of-the-art method in [22] was employed to extract the ENF signal from the video. Then the ENF signal was fed to the network to make the prediction. Note that motion detection was not applied in [22]. To yield a fair comparsion, the result in Table 4 is presented without motion detection preprocessing. As Table 4 shows, using the row luminance signal outperforms that of the ENF signal significantly, indicating that the network is able to spot the anamoly from the row luminance signal more reliably than the ENF signal. This might be due to the loss of information in ENF estimation. In addition, high-quality ENF estimation can hardly be achieved in complicated videos for real-world scenarios, making it a suboptimal option.

In addition, we also demonstrated the effectiveness of the proposed motion detection preprocessing. For this purpose, we omitted the preprocessing step for dynamic videos in both the training and testing datasets, treating them the way as static videos for subsequent processing. Both sets of experiments were conducted on the CNN-Bi-GRU-Attention model. Table 5 demonstrates that the implementation of motion detection preprocessing remarkably enhances detection performance compared to the scenario without preprocessing, leading to over 8% improvement of detection accuracy. This result also highlights the critical importance of preprocessing in dynamic video forensics.

Finally, we conducted performance comparison with the state-of-the-art scheme in [25], which is based on checking phase continuity of the ENF signal without the aid of reference ENF. The result is presented in the last row of Table 6, where the accuracy is computed at the threshold of EER. Note that the ENF signal is enhanced with our motion detection preprocessing. It was observed that our proposed method outperforms the conventional scheme by a large margin, showing the effectiveness of representation learning with deep-learning models and the bidirectional way for anomaly detection.

## 5. Conclusions

In this paper, we introduce a novel approach based on one-dimensional neural networks to detect inter-frame tampering in CMOS videos incorporating ENF signals for the first time. Instead of analyzing the ENF signal for tampering detection, which requires robust ENF estimation from video signals, our method has verified the possibility of directly applying the row luminance signal for video forensics. Utilizing the exposure characteristics of CMOS videos, we constitute a one-dimensional time series and turn video tampering detection into time series anomaly detection. In addition, a network that synergizes 1D-CNN with Bi-GRU is proposed to achieve the task of inter-frame video tampering detection. To alleviate the effect of dynamic objects in the scene, a motion detection preprocessing that greatly improves its detection performance on dynamic videos is presented. Through a series of cross-dataset experiments, the potential of our proposed model for the task of video inter-frame tampering detection under different power grids and various scenes is demonstrated.

## Figures and Tables

**Table 1:** Detailed structure of CNN-Bi-GRU-Attention model.

Layer	Output Shape	Implementation Details
Conv1	[32, 60000]	kernel size 3, stride 1
Conv2	[64, 30000]	kernel size 3, stride 1
Conv3	[128, 15000]	kernel size 3, stride 1
GRU	[7500, 128]	bidirectional, 64 hidden states
Linear	[7500, 1]	
Attention	128	
Dropout	128	ratio = 0.5
Output	2	

**Table 2:** Configurations of video datasets.

Dataset	Positive Samples (Label = 1)	Negative Samples (Label = 0)	Spatial Resolution
Dataset-1	3000	3000	640×480
Dataset-2	2000	2000	640×480

**Table 3:** Comparison of model performance with and without attention mechanism.

Model	Accuracy [%]
CNN-Bi-GRU	88.6
CNN-Bi-GRU-Attention	93.75

**Table 4:** Comparison of different inputs.

Input	Accuracy [%]
ENF signal	77.62
row luminance signal	85.56

**Table 5:** Comparison of performance with and without motion detection preprocessing.

Motion Detection Preprocessing	Accuracy [%]
w/o Preprocessing	85.56
with Preprocessing	93.75

**Table 6:** Comparison of different methods.

Method	Accuracy [%]
Proposed	93.75
Phase Continuity (non-DL) [25]	78.2

## Data Availability

The data presented in this study are available on request from the corresponding author.

## References

[1] Grigoras C. (2005). Digital audio recording analysis: The Electric Network Frequency (ENF) Criterion. Int. J. Speech Lang. Law.

[2] Brixen E.B. (2007). Techniques for the Authentication of Digital Audio Recordings. Audio Engineering Society Convention.

[3] El Gemayel T. (2013). Feasibility of Using Electrical Network Frequency Fluctuations to Perform Forensic Digital Audio Authentication. Master’s Thesis.

[4] Zheng L., Zhang Y., Lee C.E., Thing V.L.L. (2017). Time-of-recording estimation for audio recordings. Digit. Investig..

[5] Vatansever S., Dirik A.E., Memon N. (2022). ENF based robust media time-stamping. IEEE Signal Process. Lett..

[6] Bang W., Yoon J.W. (2019). Power Grid Estimation Using Electric Network Frequency Signals. Secur. Commun. Netw..

[7] Hajj-Ahmad A., Garg R., Wu M. (2015). ENF-based region-of-recording identification for media signals. IEEE Trans. Inf. Forensics Secur..

[8] Suresha P.B., Nagesh S., Roshan P.S., Gaonkar P.A., Meenakshi G.N., Ghosh P.K. (2017). A high resolution ENF based multi-stage classifier for location forensics of media recordings. Proceedings of the 2017 Twenty-third National Conference on Communications (NCC).

[9] Rodríguez D.P.A.A., Apolinário J.A., Biscainho L.W.P. (2010). Audio authenticity: Detecting ENF discontinuity with high precision phase analysis. IEEE Trans. Inf. Forensics Secur..

[10] Esquef P.A.A., Apolinário J.A., Biscainho L.W.P. (2014). Edit detection in speech recordings via instantaneous electric network frequency variations. IEEE Trans. Inf. Forensics Secur..

[11] Lin X., Kang X. (2017). Supervised audio tampering detection using an autoregressive model. Proceedings of the IEEE International Conference on Acoustics, Speech and Signal Processing (ICASSP).

[12] Wang Z.-F., Wang J., Zeng C.-Y., Min Q.-S., Tian Y., Zuo M.-Z. (2018). Digital audio tampering detection based on ENF consistency. Proceedings of the International Conference on Wavelet Analysis and Pattern Recognition (ICWAPR).

[13] Zeng C., Kong S., Wang Z., Li K., Zhao Y., Wan X., Chen Y. (2024). Digital audio tampering detection based on spatio-temporal representation learning of electrical network frequency. Multimed. Tools Appl..

[14] Hatami M., Dorje L., Li X., Chen Y. (2025). Electric Network Frequency as Environmental Fingerprint for Metaverse Security: A Comprehensive Survey. Computers.

[15] Hatami M., Qu Q., Chen Y., Mohammadi J., Blasch E., Ardiles-Cruz E. (2025). ANCHOR-Grid: Authenticating Smart Grid Digital Twins Using Real-World Anchors. Sensors.

[16] Garg R., Varna A.L., Wu M. “Seeing” ENF: Natural time stamp for digital video via optical sensing and signal processing. Proceedings of the 19th ACM International Conference on Multimedia.

[17] Garg R., Varna A.L., Hajj-Ahmad A., Wu M. (2013). “Seeing” ENF: Power-signature-based timestamp for digital multimedia via optical sensing and signal processing. IEEE Trans. Inf. Forensics Secur..

[18] Wong C.-W., Hajj-Ahmad A., Wu M. (2018). Invisible geo-location signature in a single image. Proceedings of the IEEE International Conference on Acoustics, Speech and Signal Processing (ICASSP).

[19] Choi J., Wong C.-W. (2019). ENF signal extraction for rolling-shutter videos using periodic zero-padding. Proceedings of the ICASSP 2019—2019 IEEE International Conference on Acoustics, Speech and Signal Processing (ICASSP).

[20] Choi J., Wong C.-W., Su H., Wu M. (2023). Analysis of ENF signal extraction from videos acquired by rolling shutters. IEEE Trans. Inf. Forensics Secur..

[21] Ferrara P., Sanchez I., Draper-Gil G., Junklewitz H., Beslay L. (2021). A MUSIC spectrum combining approach for ENF-based video timestamping. Proceedings of the International Workshop on Biometrics and Forensics (IWBF).

[22] Han H., Jeon Y., Song B.-k., Yoon J.W. (2022). A phase-based approach for ENF signal extraction from rolling shutter videos. IEEE Signal Process. Lett..

[23] Ngharamike E., Ang L.M., Seng K.P., Wang M. (2023). ENF based digital multimedia forensics: Survey, application, challenges and future work. IEEE Access.

[24] Nagothu D., Chen Y., Aved A.J., Blasch E. (2021). Authenticating Video Feeds using Electric Network Frequency Estimation at the Edge. EAI Endorsed Trans. Secur. Saf..

[25] Wang Y., Hu Y., Liew A.W.C., Li C.T. (2020). ENF based video forgery detection algorithm. Int. J. Digit. Crime Forensics (IJDCF).

[26] Xu L., Hua G., Zhang H., Yu L., Qiao N. “Seeing” Electric Network Frequency From Events. Proceedings of the IEEE/CVF Con-ference on Computer Vision and Pattern Recognition.

[27] Xu L., Hua G., Zhang H., Yu L. (2024). “Seeing” ENF From Neuromorphic Events: Modeling and Robust Estimation. IEEE Trans. Pattern Anal. Mach. Intell..

[28] Nagothu D., Xu R., Chen Y., Blasch E., Aved A. (2022). Defakepro: Decentralized deepfake attacks detection using enf authentication. IT Prof..

[29] Zivkovic Z. Improved adaptive Gaussian mixture model for background subtraction. Proceedings of the 17th International Conference on Pattern Recognition, ICPR.

[30] Mikolov T., Karafiát M., Burget L., Černocký J., Khudanpur S. Recurrent neural network based language model. Proceedings of the 11th Annual Conference of the International Speech Communication Association (INTERSPEECH 2010).

[31] Chung J., Gulcehre C., Cho K.H., Bengio Y. (2014). Empirical evaluation of gated recurrent neural networks on sequence modeling. arXiv.

[32] Vaswani A., Shazeer N., Parmar N., Uszkoreit J., Jones L., Gomez A.N., Kaiser Ł., Polosukhin I. Attention is all you need. Proceedings of the Advances in neural information Processing Systems 30.

[33] (2019). FFmpeg Developers, FFmpeg. https://ffmpeg.org.

[34] Hochreiter S., Schmidhuber J. (1997). Long Short-Term Memory. Neural Comput..

[35] Graves A., Schmidhuber J. (2005). Framewise phoneme classification with bidirectional LSTM and other neural network architectures. Neural Netw..

